# Cardiovascular disease and cumulative incidence of cognitive impairment in the Health and Retirement Study

**DOI:** 10.1186/s12877-021-02191-0

**Published:** 2021-04-26

**Authors:** Allyson L. Covello, Leora I. Horwitz, Shreya Singhal, Caroline S. Blaum, Yi Li, John A. Dodson

**Affiliations:** 1 New York University Grossman School of Medicine, 550 First Avenue, New York, NY USA; 2 Division of Healthcare Delivery Science, Department of Population Health, NYU Grossman School of Medicine, New York, NY USA; 3 NYU Steinhardt School of Culture, Education, and Human Development, New York, NY USA; 4 National Committee for Quality Assurance, Washington, D.C., USA; 5 Division of Biostatistics, Department of Population Health, NYU Grossman School of Medicine, New York, NY USA; 6 Leon H. Charney Division of Cardiology, Department of Medicine, NYU Grossman School of Medicine, New York, NY USA

**Keywords:** Cardiovascular disease, Cognition, Geriatric cardiology

## Abstract

**Background:**

We sought to examine whether people with a diagnosis of cardiovascular disease (CVD) experienced a greater incidence of subsequent cognitive impairment (CI) compared to people without CVD, as suggested by prior studies, using a large longitudinal cohort.

**Methods:**

We employed Health and Retirement Study (HRS) data collected biennially from 1998 to 2014 in 1305 U.S. adults age ≥ 65 newly diagnosed with CVD vs. 2610 age- and gender-matched controls. Diagnosis of CVD was adjudicated with an established HRS methodology and included self-reported coronary heart disease, angina, heart failure, myocardial infarction, or other heart conditions. CI was defined as a score < 11 on the 27-point modified Telephone Interview for Cognitive Status. We examined incidence of CI over an 8-year period using a cumulative incidence function accounting for the competing risk of death.

**Results:**

Mean age at study entry was 73 years, 55% were female, and 13% were non-white. Cognitive impairment developed in 1029 participants over 8 years. The probability of death over the study period was greater in the CVD group (19.8% vs. 13.8%, absolute difference 6.0, 95% confidence interval 2.2 to 9.7%). The cumulative incidence analysis, which adjusted for the competing risk of death, showed no significant difference in likelihood of cognitive impairment between the CVD and control groups (29.7% vs. 30.6%, absolute difference − 0.9, 95% confidence interval − 5.6 to 3.7%). This finding did not change after adjusting for relevant demographic and clinical characteristics using a proportional subdistribution hazard regression model.

**Conclusions:**

Overall, we found no increased risk of subsequent CI among participants with CVD (compared with no CVD), despite previous studies indicating that incident CVD accelerates cognitive decline.

**Supplementary Information:**

The online version contains supplementary material available at 10.1186/s12877-021-02191-0.

## Background

Cardiovascular disease (CVD) is a common chronic condition that disproportionately affects older adults [1]. Prior studies have shown a connection between CVD and cognitive impairment [2–8], with multiple potential mechanisms at play, including atherosclerotic-related cerebrovascular disease (causing cerebral hypoxia, brain infarctions, and damage to the blood-brain barrier) [9–11]; oxidative stress and inflammatory immune responses [12, 13]; and thromboembolism from concomitant atrial fibrillation [14, 15]. More recently, several longitudinal studies have indicated that incident CVD is associated with an acceleration in the decline in memory and processing speed that occurs normally with age [16–18]. This research suggests that onset of CVD may serve as a cognitive ‘inflection point,’ whereby incident CVD leads to a more rapid deterioration in patients’ cognition. However, non-significant associations have also been reported [19–21], and inconsistencies in definition of CVD, cognitive assessment tools, and follow-up duration complicate reaching generalized conclusions across studies.

Further understanding of the relationship between CVD and cognitive impairment is important in informing targeted screening and prevention efforts for cognitive impairment in vulnerable subpopulations of older adults. As such, we designed our present study to examine whether cognitively normal older adults with a diagnosis of CVD experienced a greater incidence of subsequent cognitive impairment, measured using an easily administered global assessment of cognitive function, compared to people without a diagnosis of CVD. We used data from the Health and Retirement Study (HRS), a large, well-characterized cohort study with a long duration of follow-up, an important feature given that many of the proposed biologic mechanisms connecting CVD and cognitive impairment are slow-acting processes. In order to limit the potential for bias from to high rates of attrition due to death, we used a cumulative incidence function to analyze the likelihood of cognitive impairment while accounting for the competing risk of death.

## Methods

### Data

We used data from the 1998–2014 waves of HRS. HRS is a longitudinal panel study that surveys 20,000+ Americans, nearly all of whom are age 50 or older, every 2 years [22]. The HRS survey population is largely representative of the U.S., with slight oversampling of Black, Hispanic, and Floridian households. Questions address physical health and mental health, as well as demographic characteristics like age, marital status, and level of education.

### Definition of CVD group

To define the diagnosis of CVD, we used participant self-report based on the question, “Has a doctor told you that you have had a heart attack, coronary heart disease, angina, congestive heart failure, or other heart problems?” This definition of CVD encompassed the non-stroke cardiovascular conditions, such as heart failure, myocardial infarction, atrial fibrillation, and coronary artery disease, shown in prior research to impact cognitive function [4, 6, 7, 16, 18]. The year of CVD diagnosis was defined as the first survey wave that a participant responded ‘yes’ to this question, after having responded ‘no’ in previous waves. To adjust for the potential inconsistencies in self-reported data from longitudinal surveys, we employed an adjudication method previously developed for refining participants’ responses to HRS questions about chronic disease, including CVD [23]. We then excluded anyone from the control group who was missing important demographic or cognition data, who had inconsistencies in their age reporting survey to survey, or who had cognitive impairment at or before baseline. We also excluded participants who were less than 65 at baseline, since the tool we used to assess cognitive status was not validated in a population under 65, as well as participants who were greater than 85 years old to enable appropriate generation of a matched control group per below.

### Definition of the control group

To develop a comparator control group (without CVD), we needed to define a ‘baseline’ year for control participants that could be matched to the ‘CVD diagnosis’ year for our CVD group. Because our participants diagnosed with CVD during the study period had different years of diagnosis, we used an age-matching control-generation process that pulled control participants that were the same age as CVD participants in their year of diagnosis and defined that year as ‘baseline’ for the control participants. We generated the control at a 2:1 ratio and also matched on gender to compensate for the fact that the overall HRS population skewed more female than the CVD population. We used the nearest neighbor matching methodology to generate the age-and gender-matched control, instead of an exact matching methodology, to account for the fact that matching to an exact year was not possible in the 80+ age range. As we did with our CVD group, we excluded anyone from the control group who was missing important demographic or cognition data, who had inconsistencies in their age reporting survey to survey, who was less than 65 or greater than 85 years old at baseline, or who had cognitive impairment at or before baseline.

When we completed the match, there was no significant difference in age and gender at baseline between our CVD and control groups, confirming that our matching process was successful. The complete participant selection process, including the adjudication method for CVD, the method for generating a matched control group, and the individuals removed from our final study sample, is further described in the Supplementary Methods and in Supplementary Figure S1.

### Assessment of cognitive status

The presence of cognitive impairment was determined using a modified version of the Telephone Interview for Cognitive Status (TICS-m). The TICS-m is a validated cognitive screening tool based on the Mini-Mental State Examination that has been used in prior research on the connection between CVD and cognitive impairment [5]. Unlike the Mini-Mental State Examination, the TICS-m does not need to be administered in person. The TICS-m used in HRS includes questions about immediate and delayed word recall to assess memory, serial seven subtraction to assess working memory, and counting backwards to assess information processing speed. It has high sensitivity and specificity for cognitive impairment in older adults [24, 25] and is measured on a 27-point scale, where a score of 11 or lower indicates cognitive impairment [26]. Onset of cognitive impairment was marked in the first survey wave that a participant had a TICS-m score of 11 or lower.

Some survey respondents in a given wave could not participate in the interview due to physical or mental limitations and instead used a proxy respondent. For those respondents, HRS offered an alternative measure of cognitive status using information from the proxy and the interviewer as to the interviewee’s cognitive status [27]. To avoid preferentially excluding cognitively impaired participants, who are more likely to have missing TICS values, we used the Langa-Weir cognition data from the HRS Survey Research Center, which has imputed cognition values for missing responses and is the standard dataset used for HRS cognitive assessments [28]. Additional information on cognitive assessment tools is provided in the Supplementary Methods.

### Covariates

Covariates were defined at baseline and included race and ethnicity, years of education, marital status, body mass index, current smoker, riskiness of drinking behavior, presence of depressive symptoms, and comorbid chronic conditions (hypertension, cancer, chronic lung disease, diabetes). These covariates were determined based on prior literature examining the association between CVD and cognitive impairment [16–18] and clinical judgement of study investigators.

### Statistical analysis

To examine the incidence of cognitive impairment in the CVD vs. control groups, we used a cumulative incidence function, which is a type of time-to-event analysis that looks at the probability of occurrence of an event of interest over a defined period of time [29]. Because our population was compromised of older adults, many of whom had multiple chronic health conditions, we incorporated into our analysis the competing risk of death, which takes into account the fact that death precludes our event of interest from ever occurring. This prevents the overestimation of probability of event occurrence that can happen when death is not accounted for [30]. We also calculated a proportional subdistribution hazard regression model to adjust for covariates while simultaneously accounting for competing risk [29].

Additionally, we performed 2 sensitivity analyses. In the first sensitivity analysis, we redefined our exclusion criteria to be less stringent and included in the CVD group participants previously excluded due to self-report discrepancies. We then generated a new matched control and repeated the cumulative incidence function with the new CVD and control groups. In the second, we redefined the threshold for cognitive impairment using TICS-m cutoffs of 10 and 12 instead of 11 and reran the cumulative incidence function with the new thresholds.

All data cleaning and analyses were conducted using RStudio (RStudio, Inc. 2020) [31]. The cumulative incidence function was generated using the cmprsk package [32]. All analyses used publicly available, non-restricted data collected through the Health and Retirement Survey and did not require IRB/human subjects review.

## Results

### Baseline characteristics and sample size

**Fig. 1:**
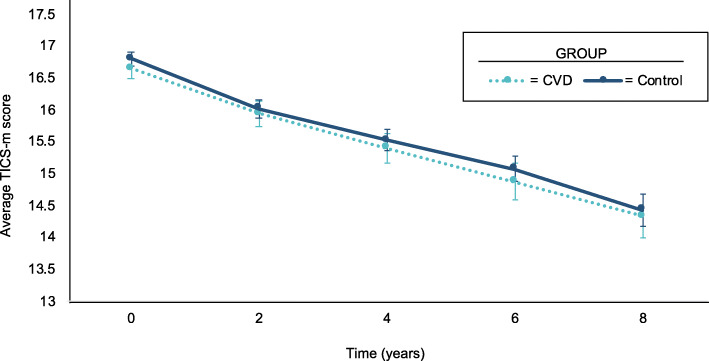
Trend in Mean Modified Telephone Interview for Cognitive Status Score Over Time. Shown are the mean TICS-m scores in the CVD group (dashed light blue line) vs. control group (solid dark blue line) over the 8-year study period. Bars represent 95% confidence intervals

The final sample consisted of 1305 participants with incident CVD and 2610 age- and gender-matched controls. Mean age at study entry was 73 years, and 55% were female. 87% of participants were non-Hispanic white, 5% were Hispanic, and 7% were non-Hispanic Black (Table 1). Mean length of follow-up was 5.01 years (standard deviation = 1.49) in the incident CVD group and 5.33 years (standard deviation = 1.35) in the control group. The mean TICS-m scores at baseline were not significantly different between the incident CVD and control groups (16.6, 95% confidence interval 16.5 to 16.8 vs. 16.8, 95% confidence interval 16.7 to 16.9), and trended downwards at the same rate during the 8-year study period (Fig. 1). The TICS-m scores for CVD and control groups had similar distributions at baseline and at years 2, 4, 6 and 8 (Supplementary Figure S2).

**Table 1:** Baseline Sample Size and Cohort Characteristics

Variable	Incident CVD Group ***n*** = 1305	Matched Control Group ***n*** = 2610
Age (yrs)	73.3 (5.63)	73.3 (5.64)
Women	702 (53.8%)	1455 (55.7%)
Race & Ethnicity		
Non-Hispanic White	1170 (89.7%)	2218 (85.0%)
Non-Hispanic Black	69 (5.3%)	193 (7.4%)
Hispanic	50 (3.8%)	154 (5.9%)
Other	16 (1.2%)	45 (1.7%)
Education level		
Less than high school	179 (13.7%)	381 (14.6%)
High school	478 (36.6%)	952 (36.5%)
More than high school	648 (49.7%)	1277 (48.9%)
BMI (kg/m^2)	27.4 (5.54)	26.7 (4.75)
Married	819 (62.8%)	1684 (64.5%)
Depressive symptoms	163 (12.5%)	207 (7.9%)
Smoking currently	102 (7.8%)	237 (9.1%)
Drinking behavior (past 3 mos)		
Not drinking	891 (68.3%)	1574 (60.3%)
Low-risk drinker	402 (30.8%)	1015 (38.9%)
High-risk drinker	12 (0.9%)	21 (0.8%)
Hypertension	927 (71.0%)	1431 (54.8%)
Diabetes	324 (24.8%)	400 (15.3%)
Chronic lung disease	182 (13.9%)	205 (7.9%)
Cancer	278 (21.3%)	515 (19.7%)
TICS-m score	16.6 (2.87)	16.8 (2.91)

Values are mean + − standard deviation or n (%). Race and ethnicity is defined based on participants’ primary response, and ‘other’ for race and ethnicity is defined as American Indian, Alaska Native, Asian, Native Hawaiian, Pacific Islander, or something else. High-risk drinking is defined as more than 7 drinks per week or 3 drinks per day for women, or more than 14 drinks per week or 4 drinks per day for men on average over the past 3 months

*Abbreviations*: *CVD* cardiovascular disease, *BMI* body mass index, *TICS-m* modified Telephone Interview for Cognitive Status

The number of participants at risk of cognitive impairment in the CVD group was 1305 (100%), 1062 (81%), 769 (59%), and 487 (37%) at 2, 4, 6, and 8 years. The number of participants at risk of cognitive impairment in the control group was 2610 (100%), 2182 (84%), 1570 (60%), and 1054 (40%) at 2, 4, 6, and 8 years.

### Cumulative incidence of cognitive impairment and death

**Fig. 2:**
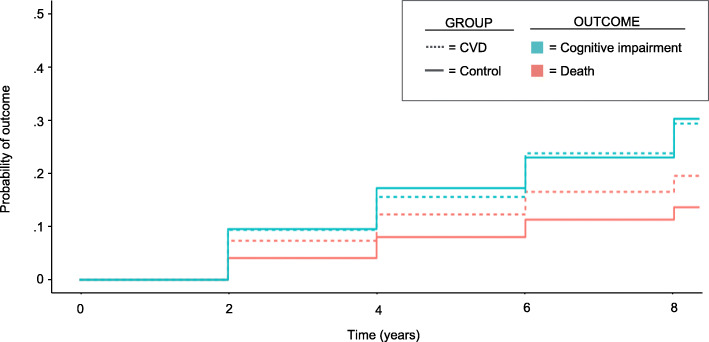
Cumulative Incidence of Cognitive Impairment in CVD vs. Control Groups. Shown are the likelihood of death (orange) and the likelihood of cognitive impairment (blue) in the CVD group (dashed line) vs. control group (solid line) over the 8-year study period. Participants with CVD were more likely to experience death at follow-up than controls. There was no significant difference in the incidence of cognitive impairment between participants with CVD vs. controls

We examined the cumulative incidence of cognitive impairment in the CVD and control groups over the 8-year study period, accounting for the competing risk of death. During the study period, cognitive impairment developed in 339 (26.0%) of participants in the CVD group and 690 (26.4%) of participants in the control group. Death before cognitive impairment occurred in 232 (17.8%) of participants in the CVD group and 317 (12.1%) of participants in the control group. Per the cumulative incidence analysis, the probability of death over 8 years was greater in the CVD group (19.8% vs. 13.8%, absolute difference 6.0, 95% confidence interval 2.2 to 9.7%). The probability of cognitive impairment accounting for the competing risk of death over 8 years was not significantly different between the CVD and control groups (29.7% vs. 30.6%, absolute difference − 0.9, 95% confidence interval − 5.6 to 3.7%) (Fig. 2).

### Subdistribution hazard regression model of incidence of cognitive impairment and death

Given the potential effect of covariates on the cumulative incidence of cognitive impairment, we created a proportional subdistribution hazard regression model, which assesses the effect of multiple variables on incidence of a particular event in a competing risk analysis. The subdistribution hazard ratios and their associated 95% confidence intervals describe whether a particular variable has a significant effect on the outcome of interest, such as cognitive impairment, and the direction of this relationship (i.e. increases or decreases outcome incidence), though the magnitude of the ratio cannot be inferred as representing the relative increase in incidence of the outcome of interest [33].

Based on our proportional subdistribution hazard regression model, when incorporating covariates of race and ethnicity, education, body mass index, marital status, depressive symptoms, current smoking, level of alcohol intake, and chronic conditions (hypertension, diabetes, chronic lung disease, and cancer) into the analysis, incident CVD remained not significant in prognosticating incidence of cognitive impairment (subdistribution hazard ratio = .96, 95% confidence interval .85 to 1.09). The subdistribution hazard ratios and 95% confidence intervals for each variable in the model are further outlined in Table 2.

**Table 2:** Effect of CVD and Covariates on Incidence of Cognitive Impairment per Subdistribution Hazard Regression Model

Variable	Subdistribution Hazard Ratio (95% Confidence Interval)
Incident CVD	0.96 (0.85–1.09)
Age (yrs)	1.06 (1.05–1.07)
Male gender	1.10 (0.97–1.25)
Race & Ethnicity	
Non-Hispanic Black	1.79 (1.47–2.18)
Hispanic	1.33 (1.05–1.67)
Other	1.45 (0.93–2.28)
Education level	
Less than high school	1.82 (1.55–2.15)
High school	1.38 (1.21–1.58)
BMI (kg/m^2)	0.99 (0.97–1.00)
Married	0.965 (0.88–1.14)
Depressive symptoms	1.23 (1.03–1.48)
Smoking currently	1.18 (0.95–1.44)
Drinking behavior (past 3 mos)	
Not drinking	0.909 (0.52–1.62)
Low-risk drinker	0.67 (0.39–1.23)
Hypertension	1.05 (0.92–1.19)
Diabetes	1.01 (0.87–1.19)
Chronic lung disease	0.94 (0.76–1.15)
Cancer	0.94 (0.81–1.10)

Race and ethnicity is defined based on participants’ primary response, and ‘other’ for race and ethnicity is defined as American Indian, Alaska Native, Asian, Native Hawaiian, Pacific Islander, or something else. Low-risk drinking is defined as 7 drinks or fewer per week or 3 drinks or fewer per day for women, or 14 drinks or fewer per week or 4 drinks or fewer per day for men on average over the past 3 months

*Abbreviations*: *CVD* cardiovascular disease, *BMI* body mass index

### Sensitivity analyses

One hundred eighty-nine participants who met other inclusion criteria (not cognitively impaired before CVD diagnosis, not missing key cognition or covariate data, and between 65 and 85 at time of CVD onset) were excluded from the main analysis due to unresolved discrepancies in CVD self-report. These 189 participants with self-report discrepancies were more likely to develop cognitive impairment during the study period (41.3% vs. 26.0%) and less likely to experience death before cognitive impairment (7.9% vs. 17.8%) (Supplementary Table S1). We then re-examined the cumulative incidence of cognitive impairment in the CVD and control groups accounting for the competing risk of death, including these 189 participants. This new sample consisted of 1494 participants with incident CVD and 2988 age- and gender-matched controls. Per the cumulative incidence analysis, the probability of death over 8 years was still greater in the CVD group than in the control group (17.9% vs. 13.3%, absolute difference 4.6, 95% confidence interval 1.1 to 7.9%). The probability of cognitive impairment accounting for the competing risk of death over 8 years was still not significantly different between the CVD and control groups (31.7% vs. 29.9%, absolute difference 1.8, 95% confidence interval − 2.4 to 6.2%) (Supplementary Figure S3).

We performed an additional sensitivity analysis to examine the effects of altering the TICS-m score cutoff for cognitive impairment on the cumulative incidence of cognitive impairment in the CVD vs. control groups. Defining cognitive impairment as a TICS-m score of 12 or below, the probability of cognitive impairment over the 8-year study period increased to 38.9% in the CVD group (vs 29.7% in the original analysis) and 37.3% in the control group (versus 30.6% in the original analysis). Defining cognitive impairment as a TICS-m score of 10 or below, the probability of cognitive impairment over the 8-year study period decreased to 22.0% in the CVD group (versus 29.7% in the original analysis) and 20.5% in the control group (versus 30.6% in the original analysis). The probability of cognitive impairment accounting for the competing risk of death over 8 years was not significantly different between the CVD and control groups with a TICS-m cutoff of 12 (absolute difference 1.6, 95% confidence interval − 3.1 to 6.0%) or with a TICS-m cutoff of 10 (absolute difference 1.5, 95% confidence interval − 3.7 to 6.9%) (Supplementary Table S2).

## Discussion

In a large longitudinal study of older adults (mean age 73 years), we observed an increased probability of death but no increased probability of cognitive impairment among participants with incident CVD compared with an age- and gender-matched control group. This finding contradicts several recent studies indicating that incident CVD accelerates cognitive decline [16–18]. However, there are several key differences between our study and previous studies that may account for our results. Firstly, our study utilized a cumulative incidence analysis accounting for the competing risk of death, which can be thought of as a prognostic model that examines the probability of cognitive impairment occurring in CVD vs. control patients in a real-world setting where death is a possibility [30]. This type of prognostic model that aims to predict likelihood is different than an etiological analysis that aims to infer the causal relationship between CVD and cognitive impairment. One logical interpretation of our results is that higher rates of death may preclude the development of cognitive impairment in a significant portion of the CVD population, as these participants may not live long enough after CVD diagnosis to develop significant cognitive decline. This is consistent with the idea that many of the proposed biologic mechanisms leading to cognitive impairment in CVD patients, such as systemic inflammation, oxidative damage, and subclinical vascular brain injury, are slow-acting processes that take many years to occur [14, 17].

Additionally, in measuring cognitive impairment, our study used a modified version of the TICS (TICS-m), which is a validated cognitive screening tool based on the Mini-Mental State Examination. While the TICS-m has high sensitivity and specificity for cognitive impairment in older adults [24, 25], it is designed to screen for cognitive impairment and does not provide the same degree of granularity as measuring specific domains of cognitive function, such as verbal memory, information processing speed, and temporal orientation, separately. Our results support the notion that CVD patients are not more likely than their non-CVD counterparts to experience global cognitive changes (that would clinically manifest as mild cognitive impairment or dementia) but do not speak to whether CVD patients have declines in cognitive performance in specific domains. Multiple previous studies examining accelerated cognitive decline in CVD patients have examined isolated cognitive domains rather than global cognitive status, and these studies have observed that CVD patients have faster cognitive decline in certain domains, such as verbal memory, information processing speed, and temporal orientation, but not in others, such as executive function and semantic fluency [17, 18]. It is difficult to extrapolate whether these observed declines in specific cognitive domains would translate into global cognitive impairment that manifests clinically.

While the weight of prior evidence has suggested a connection between CVD and cognitive impairment, these findings are not universal. A meta-analysis conducted in 2017 found that taken together, prospective cohort studies showed increased risk of cognitive impairment in individuals with coronary heart disease, defined as individuals with angina pectoris and myocardial infarction, while cross-sectional and case-control studies did not [3]. Another review that analyzed longitudinal studies on cognitive impairment with atrial fibrillation, heart failure, peripheral artery disease, myocardial infarction, and impact of atherosclerotic burden separately found that atrial fibrillation and severe atherosclerosis were risk factors for cognitive decline but that the body of literature on heart failure, peripheral artery disease, and myocardial infarction was too small to draw any conclusions [8]. There is also significant heterogeneity across the literature in how CVD and cognitive impairment are defined and measured. Since CVD is a general term that can be comprised of many cardiovascular-related conditions (e.g. myocardial infarction, coronary artery disease, heart failure, arrythmia, stroke, etc.), studies have included different conditions in their analyses. Cognitive impairment is also a very broad term that can be defined and segmented in different ways (e.g. mild cognitive impairment vs. dementia, non-amnesiac cognitive impairment vs. amnesiac cognitive impairment), or examined by looking at domains of cognition (e.g. verbal memory, information processing speed, temporal orientation). This methodological heterogeneity makes it difficult to draw broader conclusions from the current body of literature. Further, it is also important to consider that bias towards publishing positive results may have played a role in shaping the current literature landscape.

Our study has several strengths, including a well-characterized dataset with a large sample size and serial cognitive assessments over nearly a decade of follow-up. Our study also has several important limitations. First, we relied on self-reported CVD. While we used an established HRS methodology to apply rigor to this process, we also found that participants with inconsistencies in self-report were more likely to have cognitive impairment, suggesting that cognitive status did play a role in participants’ abilities to report on their cardiovascular disease status. However, our sensitivity analysis that included participants with self-report discrepancies still showed no difference in incidence of cognitive impairment between CVD and control groups. Utilizing self-report also resulted in a broader analysis of cognitive impairment in CVD overall, as opposed to a more focused analysis looking at cognitive impairment in one type of CVD, such as heart failure or myocardial infarction. While this limited the specificity of our analysis, prior research has proposed an association between cognitive impairment and the range of cardiac conditions, such as heart failure, myocardial infarction, atrial fibrillation, and coronary artery disease, that were included in our definition of CVD [4, 6, 7, 16, 18].

A second limitation is that several variables were not available in in our dataset and may have influenced the results. For example, we lacked robust data on the severity of participants’ CVD or how they managed their symptoms. It is possible that patients with more severe atherosclerotic disease burden were more likely to develop cognitive impairment. Third, we performed a clinical cutpoint-based analysis, rather than a continuous analysis of change in TICS-m, which limited our ability to detect differences between the CVD and control groups in the rate of decline in TICS-m or the association of incident CVD with TICS-m at the time of CVD onset. We decided on a cutpoint-based analysis to ease clinical interpretability, but we acknowledge the potential loss of information in doing so. Fourth, we used the Langa-Weir cognition dataset that included imputed values for participants missing some TICS responses. We employed this data to align with prior HRS studies and to minimize bias from exclusion of cognitively impaired participants, as not using these values leads to missing a large fraction of participants with dementia. However, we recognize that the imputation may have influenced results. Fifth, our sample included primarily non-Hispanic white participants, which does not reflect the diversity of race and ethnicity present in the U.S. population and limits the generalizability of our results. Sixth, we excluded a significant portion of the HRS population with CVD due to missing covariate data and age restrictions that were used to generate a sufficient matched cohort. Seventh, we did not control for presence of the ApoE4 allele, which some past studies on CVD and cognition accounted for given its strong association with dementia [34]. Lastly, we did not have data on specific cognitive domains, so we were unable to analyze cognitive impairment at a more granular level as other studies have done.

## Conclusions

In summary, our results show an increased likelihood of death but not cognitive impairment among participants with incident CVD vs. an age- and gender-matched control group. From a clinical perspective, cognitive screening may still be useful in patients with CVD. There are many CVD patients with cognitive decline that impairs their ability to engage in self-care [35], and factors such as nonadherence to medication can lead to adverse outcomes. However, based on our findings and other negative studies [19–21], patients with CVD may not be a subgroup that requires targeted cognition screening beyond what is recommended for older adults more broadly.

## Supplementary Information

**Additional file 1: Supplementary Methods.** Definition of CVD and Cognitive Impairment. **Supplementary Table S1.** Sample Size and Cohort Characteristics of CVD Group vs. Participants Excluded due to Self-report Inconsistency. **Supplementary Table S2.** Change in Incidence of Cognitive Impairment with Varying TICS-m Score Cutoffs. **Supplementary Figure S1.** Flowchart of Participant Selection. **Supplementary Figure S2.** Distribution of Modified Telephone Interview for Cognitive Status Scores for CVD and Control Groups. **Supplementary Figure S3.** Cumulative Incidence of CI in CVD Group Including Self-report Discrepancies vs. Control Group.

## Data Availability

The datasets analyzed in this study are available in the Health and Retirement Study repository: https://hrs.isr.umich.edu/data-products?_ga=2.9793781.1130511234.1607196676-1851121076.1607196676

## Abbreviations

CVD: Cardiovascular disease
HRS: Health and retirement study
TICS-m: Modified telephone interview for cognitive status
