# Supplementary material for: Cardiovascular disease and cumulative incidence of cognitive impairment in the Health and Retirement Study
Source: BMC Geriatr. 2021 Apr 26;21:274. doi: 10.1186/s12877-021-02191-0 (PMC8074515; doi:10.1186/s12877-021-02191-0)
Supplement: Supplementary file 1 — Additional file 1: Supplementary Methods. Definition of CVD and Cognitive Impairment. Supplementary Table S1. Sample Size and Cohort Characteristics of CVD Group vs. Participants Excluded due to Self-report Inconsistency. Supplementary Table S2. Change in Incidence of Cognitive Impairment with Varying TICS-m Score Cutoffs. Supplementary Figure S1. Flowchart of Participant Selection. Supplementary Figure S2. Distribution of Modified Telephone Interview for Cognitive Status Scores for CVD and Control Groups. Supplementary Figure S3. Cumulative Incidence of CI in CVD Group Including Self-report Discrepancies vs. Control Group. [file 12877_2021_2191_MOESM1_ESM.docx]

**Cardiovascular Disease and Cumulative Incidence of Cognitive Impairment in the Health and Retirement Study**

Allyson L. Covello BA,^1^ Leora I. Horwitz MD,^2^ Shreya Singhal MS,^3^ Caroline S. Blaum MD, ^4^ Yi Li MS, ^5^ John A. Dodson MD, MPH^2,6^

^1^New York University Grossman School of Medicine, New York, NY, USA

^2^Division of Healthcare Delivery Science, Department of Population Health, NYU Grossman School of Medicine, New York, NY, USA

^3^NYU Steinhardt School of Culture, Education, and Human Development, New York, NY, USA

^4^National Committee for Quality Assurance, Washington, D.C., USA

^5^Division of Biostatistics, Department of Population Health, NYU Grossman School of Medicine, New York, NY, USA

^6^Leon H. Charney Division of Cardiology, Department of Medicine, NYU Grossman School of Medicine, New York, NY, USA

**SUPPLEMENTAL MATERIALS**

**Supplementary Methods: Definition of CVD and Cognitive Impairment**

*Definition of CVD Group*

To adjust for the potential inconsistencies in self-reported data from longitudinal surveys, we employed an adjudication method previously developed for refining participants’ responses to HRS questions about chronic disease, including CVD [23]. This method involved looking at participants’ responses to additional CVD questions regarding medication use, previous surgery, and what specific heart condition they had. These questions were seen as further support for a participant having CVD and were used to resolve discrepancies where respondents who had indicated that they had CVD reported in the subsequent survey wave that they did not have CVD.

After applying this adjudication method to resolve survey-to-survey discrepancies, we then excluded any participants who still had self-report inconsistencies over time or who were missing CVD data the year before they responded ‘yes’ to the survey question about CVD, as we could not accurately determine their year of CVD diagnosis. Participants who reported CVD in the first year they were surveyed were classified as having prevalent CVD and were also excluded from the main analysis, as we could not determine year their year of CVD diagnosis, and our question of interest was in describing cognitive impairment in the years following incident CVD. We further excluded participants with cognitive impairment in the year of or before CVD diagnosis, using any of the following: (1) abnormal cognitive testing (described below); (2) self-reported diagnosis of Alzheimer’s disease or other dementia; or (3) use of medication prescribed by a doctor to help with memory problems. This exclusion was necessary to ensure that participants were not cognitively impaired at the time of CVD diagnosis. We also excluded any HRS participants under the age of 65, as our cognitive assessment tool (described below) had not been extensively validated in this younger population, and any participants over the age of 85, as we could not appropriately generate an age-matched control group for these older participants. Additional exclusion criteria included missing or inconsistent age data in any survey year, missing gender/education data in all survey years missing data on cognition at baseline or during 8-year follow-up period, and missing data on marital status, BMI, smoking status, riskiness of drinking behavior, presence of depressive symptoms, or comorbid chronic conditions (hypertension, cancer, chronic lung disease, and diabetes) at baseline (aka year of CVD onset).

*Definition of Control Group*

We used an age-matching control-generation process that pulled control participants that were the same age as CVD participants in their year of diagnosis and defined that year as ‘baseline’ for the control participants. We generated the control at a 2:1 ratio and also matched on gender to compensate for the fact that the overall HRS population skewed more female than the CVD population. We used the nearest neighbor matching methodology to generate the age-and gender-matched control, instead of an exact matching methodology, to account for the fact that matching to an exact year was not possible in the 80+ age range. As we did with our CVD group, we excluded anyone from the control group who was missing important demographic or cognition data, who had inconsistencies in their age reporting survey to survey, who were less than 65 or greater than 85 years old at baseline, or who had cognitive impairment at or before baseline.

To illustrate this control generation process further, take the example of a participant diagnosed with CVD in survey year 2000, when she was 68 years old. We included in the control group 2 participants who were also women age 68 in 2000, and we called 2000 the baseline year, or time=0, for these 3 participants. We repeated this process for every person diagnosed with CVD in 2000, then 2002, then 2004, and so forth through 2014.

*Assessment of cognitive status*

*Proxy respondents*: Some survey respondents in a given wave could not participate in the interview due to physical or mental limitations and instead used a proxy respondent. For those respondents, HRS offered an alternative measure of cognitive status using information from the proxy and the interviewer as to the interviewee’s cognitive status. This proxy cognition screener was scored on a 0-9 scale in the 1998 wave and a 0-11 scale in 2000-214 waves, where a score of 3 or greater was indicative of cognitive impairment.

*Imputed cognition values*: To further reduce the number of missing cognition data values, we used updated cognition data from the HRS Survey Research Center that had imputed cognition values for missing responses [28]. Using imputed data was important because we could not assume that cognition values were missing at random. The imputation was performed using a multivariate, regression-based procedure to replace refusals, missing values, and NA responses in the TICS-m data, but not the proxy respondent data.

*Defining year of cognitive impairment onset*: Onset of cognitive impairment was marked in the first survey wave that a participant had a TICS-m score of 11 or lower, or a proxy score of 3 or greater. We defined this as cognitive impairment onset even if a participant’s score reverted to 12 or greater in a subsequent survey year because of research showing that participants often experience slight improvement in cognitive screening scores when tests are administered on a 1-2 year time frame due to practice effects [1-3]. Additionally, a drop in cognition is clinically meaningful even if some cognitive capability is regained in the future.

**Supplementary Tables**

**Supplementary Figures**

**Supplementary Figure S1. Flowchart of Participant Selection.**

**Supplementary Figure S2: Distribution of Modified Telephone Interview for Cognitive Status Scores for CVD and Control Groups.** Shown are histograms of TICS-m scores in the CVD group (light blue) vs. control group (dark blue) at baseline and at 2, 4, 6, and 8 years.

**Supplementary Figure S3: Cumulative Incidence of CI in CVD Group Including Self-report Discrepancies vs. Control Group.** Because the participants excluded due to self-report discrepancies were more likely to develop cognitive impairment, we performed a sensitivity analysis where we included these initially excluded participants in the CVD group and re-examined the cumulative incidence of cognitive impairment in the CVD and control groups accounting for the competing risk of death. Shown are the likelihood of death (orange) and the likelihood of cognitive impairment (blue) in the CVD group (dashed line) vs. control group (solid line) over the 8-year study period. Participants with CVD were more likely to experience death at follow-up than controls. There was no significant difference in the incidence of cognitive impairment between participants with CVD vs. controls.

References

1. Cooley SA, Heaps JM, Bolzenius JD, et al. Longitudinal Change in Performance on the Montreal Cognitive Assessment in Older Adults. *Clin. Neuropsychol.* 2015;29(6):824–835.

2. Hensel A, Angermeyer MC, Riedel-Heller SG. Measuring cognitive change in older adults: reliable change indices for the Mini-Mental State Examination. *J. Neurol. Neurosurg. Psychiatry*. 2007;78(12):1298–1303.

3. Jacqmin-Gadda H, Fabrigoule C, Commenges D, et al. Longitudinal study of cognitive aging in non-demented elderly subjects. *Rev. Epidemiol. Sante Publique*. 1997;45(5):363–372.
